# Efficacy of an Immunotherapy Combining Immunogenic Chimeric Protein Plus Adjuvant and Amphotericin B against Murine Visceral Leishmaniasis

**DOI:** 10.3390/biology12060851

**Published:** 2023-06-13

**Authors:** Danniele L. Vale, Camila S. Freitas, Vívian T. Martins, Gabriel J. L. Moreira, Amanda S. Machado, Fernanda F. Ramos, Isabela A. G. Pereira, Raquel S. Bandeira, Marcelo M. de Jesus, Grasiele S. V. Tavares, Fernanda Ludolf, Miguel A. Chávez-Fumagalli, Alexsandro S. Galdino, Ricardo T. Fujiwara, Lílian L. Bueno, Bruno M. Roatt, Myron Christodoulides, Eduardo A. F. Coelho, Daniela P. Lage

**Affiliations:** 1 Programa de Pós-Graduação em Ciências da Saúde: Infectologia e Medicina Tropical, Faculdade de Medicina, Universidade Federal de Minas Gerais, Belo Horizonte 30130-100, Minas Gerais, Brazil; 2 Laboratório de Imunopatologia, Núcleo de Pesquisas em Ciências Biológicas/NUPEB, Departamento de Ciências Biológicas, Insituto de Ciências Exatas e Biológicas, Universidade Federal de Ouro Preto, Ouro Preto 35400-000, Minas Gerais, Brazil; 3 Computational Biology and Chemistry Research Group, Vicerrectorado de Investigación, Universidad Católica de Santa María, Urb. San José S/N, Umacollo, Arequipa 04000, Peru; 4 Laboratório de Biotecnologia de Microrganismos, Universidade Federal de São João Del-Rei, Divinópolis 35501-296, Minas Gerais, Brazil; 5 Departamento de Parasitologia, Instituto de Ciências Biológicas, Universidade Federal de Minas Gerais, Belo Horizonte 31270-901, Minas Gerais, Brazil; 6 Neisseria Research Group, Molecular Microbiology, School of Clinical and Experimental Sciences, University of Southampton Faculty of Medicine, Southampton General Hospital, Southampton SO16 6YD, UK; 7 Departamento de Patologia Clínica, Colégio Técnico (COLTEC), Universidade Federal de Minas Gerais, Av. Antônio Carlos, 6627, Belo Horizonte 31270-901, Minas Gerais, Brazil

**Keywords:** *Leishmania*, visceral leishmaniasis, immunotherapy, chimera vaccine, T cell response, mouse, monophosphoryl lipid A, amphotericin B

## Abstract

**Simple Summary:**

Visceral leishmaniasis is a tropical neglected disease caused by infection with *Leishmania* parasites. There are no human vaccines and the current drug treatments for infected patients are hampered by toxicity and the development of resistance. In our study, we have used an immunotherapeutic approach, which combines a vaccine and drug, to treat mice infected with the parasite. This involved injecting a candidate *Leishmania* vaccine ChimT with the adjuvant monophosphoryl lipid A (MPLA, which enhances the immune response) and a treatment drug amphotericin B (AmpB). We found that the combined immunotherapy of ChimT/MPLA/AmpB significantly reduced the number of parasites within infected internal organs of the animals and increased the production of protective antibodies. Organ toxicity was also lower with the ChimT/MPLA/AmpB immunotherapy, suggesting that the inclusion of the vaccine and adjuvant reduced the inherent toxicity of the drug. In addition, the ChimT vaccine stimulated in vitro mouse white cells to kill three different *Leishmania* parasites that had invaded the cells. We conclude that the combination of ChimT/MPLA/AmpB could be considered for further studies as an immunotherapy for *Leishmania* infection.

**Abstract:**

Visceral leishmaniasis (VL) in the Americas is a chronic systemic disease caused by infection with *Leishmania infantum* parasites. The toxicity of antileishmanial drugs, long treatment course and limited efficacy are significant concerns that hamper adequate treatment against the disease. Studies have shown the promise of an immunotherapeutics approach, combining antileishmanial drugs to reduce the parasitism and vaccine immunogens to activate the host immune system. In the current study, we developed an immunotherapy using a recombinant T cell epitope-based chimeric protein, ChimT, previously shown to be protective against *Leishmania infantum*, with the adjuvant monophosphoryl lipid A (MPLA) and amphotericin B (AmpB) as the antileishmanial drug. BALB/c mice were infected with *L. infantum* stationary promastigotes and later they received saline or were treated with AmpB, MPLA, ChimT/Amp, ChimT/MPLA or ChimT/MPLA/AmpB. The combination of ChimT/MPLA/AmpB significantly reduced the parasite load in mouse organs (*p* < 0.05) and induced a Th1-type immune response, which was characterized by higher ratios of anti-ChimT and anti-parasite IgG2a:IgG1 antibodies, increased IFN-γ mRNA and IFN-γ and IL-12 cytokines and accompanied by lower levels of IL-4 and IL-10 cytokines, when compared to other treatments and controls (all *p* < 0.05). Organ toxicity was also lower with the ChimT/MPLA/AmpB immunotherapy, suggesting that the inclusion of the vaccine and adjuvant ameliorated the toxicity of AmpB to some degree. In addition, the ChimT vaccine alone stimulated in vitro murine macrophages to significantly kill three different internalized species of *Leishmania* parasites and to produce Th1-type cytokines into the culture supernatants. To conclude, our data suggest that the combination of ChimT/MPLA/AmpB could be considered for further studies as an immunotherapy for *L. infantum* infection.

## 1. Introduction

Visceral leishmaniasis (VL) is a potentially fatal parasitic disease endemic in over 70 countries with an at-risk population of over 200 million people [1]. Worldwide and annually, approximately 500,000 new cases of VL are reported with 50,000 deaths, and more than 90% of these cases and deaths occur in Brazil, Ethiopia, India, Kenya, Somalia, South Sudan and the Sudan [2]. In the Americas, VL is a zoonosis caused by *Leishmania infantum* parasites, where domestic animal reservoirs, such as dogs, are relevant for their transmission cycle [3]. Furthermore, a VL diagnosis is difficult to perform, mainly due to the similarity with other more common clinical pathologies [4,5].

Chemotherapy for VL is fraught with several issues. The drugs of choice for more than 60 years have been the pentavalent antimonials, but their use is associated with high toxicity, and they are not suitable for use in children, for pregnant and breastfeeding women and for chronically ill patients [6]. Amphotericin B (AmpB) is a fungal agent also used to treat VL in Brazil, and its mechanism of action involves binding to parasite membrane sterols, which alters parasite cell permeability and causes the loss of cations and *Leishmania* death [7]. The drug has been used successfully against distinct *Leishmania* species that cause tegumentary and visceral leishmaniasis [8,9,10,11]. However, AmpB is also toxic and can induce cardiac alterations and renal and hepatic damage [12]. The expansion of Human Immunodeficiency Virus (HIV)-VL coinfection cases also has influenced the epidemiology of VL worldwide [13]. In this context, alternative therapeutic interventions are needed that can promote an effective immune response in infected hosts and improve health [14]. One possibility is immunotherapy, which involves the synergism between antileishmanial drugs that reduce and/or eliminate the parasites and immunogenic molecules that could activate a protective host immune response [15,16].

Regarding the immune response against VL, studies have showed that susceptibility to infection correlates with the depression of Th1-type immunity and low production of cytokines such as IFN-γ, IL-2 and IL-12 by both CD4^+^ and CD8^+^ T cell subtypes. In addition, the development of the disease is associated with a marked up-regulation of IL-4, IL-10 and IL-13 cytokines [17,18,19,20]. In patients with the active disease, there is a polyclonal B-cell expansion that causes specific immunosuppression, and distinct organs are infected by the parasites [21]. In this context, an immunotherapeutic candidate should be able to induce or activate a Th1-type immune response. However, despite advances in developing vaccines against canine disease, there is still no approved human vaccine [22,23]. In addition, it is uncertain whether a vaccine based on a single recombinant protein would provide protection against a complex parasite, which usually expresses hundreds of proteins [24]. One strategy to overcome the need for multiple recombinant proteins in a vaccine is the use of chimeric proteins, which contain immunogenic T cell epitopes identified from multiple *Leishmania* antigens. These chimeric antigens offer the advantages of more facile production at a lower cost and with an improved manufacturing practice, compared to the use of distinct recombinant proteins or the development of new antileishmanial drugs [25,26]. Immune responses to vaccine protein antigens are invariably increased by the incorporation of adjuvants. Effective adjuvants have been proven to increase both antigenicity and immunogenicity, reduce both the concentration of the antigen required and the number of doses to elicit protection, and ensure the development of a more robust Th1-type immune response. Monophosphoryl lipid A (MPLA) is a commonly used adjuvant that is derived from the lipopolysaccharide (LPS) of *Salmonella minnesota* Re595 [27], in which toxicity is abrogated by the elimination of phosphate and fatty acid groups from the parent LPS molecule [28]. MPLA has been shown to stimulate the mammalian immune response and preferentially induce a Th1-type response [29,30,31,32].

Recently, a recombinant chimeric protein called ChimT was constructed, expressed and purified, and evaluated as a vaccine against *L. infantum* murine infection. ChimT contains identified T cell epitopes from four *Leishmania* proteins, namely prohibitin, the eukaryotic initiation factor 5a and the hypothetical LiHyp1 and LiHyp2 proteins. In the murine model of leishmaniasis, immunization with ChimT plus saponin as the adjuvant stimulated significantly higher levels of IFN-γ, IL-12 and GM-CSF by CD4^+^ and CD8^+^ T cell subtypes, with higher production of the anti-parasite IgG2a antibody and correspondingly lower levels of IL-4 and IL-10 cytokines [33]. In addition, ChimT induced a lymphoproliferative response in peripheral blood mononuclear cells (PBMC) from VL patients after treatment and from healthy subjects, as well as higher levels of IFN-γ and lower levels of IL-10 into spleen cell culture supernatants. Notably, active prophylactic immunization with ChimT prevented parasite numbers from increasing within internal organs, compared to control mice that had a high burden of visceral parasitism [33].

In the current study, we have evaluated a new immunotherapeutic strategy that involved associating an immunogenic protein, ChimT, with MPLA as the adjuvant and AmpB as the antileishmanial drug. Our purpose was to investigate if this strategy could alter the disease profile of BALB/c mice that had been infected chronically with *L. infantum*. The mice were infected and after 60 days they received the immunotherapeutic protocols; after 1 and 30 days, respectively, they were euthanized and tissue and serum samples were used to evaluate biochemical, immunological and parasitological parameters.

## 2. Materials and Methods

### 2.1. Mice and Parasites

The Committee on the Ethical Handling of Research Animals of UFMG approved this study and provided the protocol number 144/2020. The breeding facilities of the Bioterism Center of Federal University of Minas Gerais (UFMG; Belo Horizonte, Minas Gerais, Brazil) provided female BALB/c mice of 8 weeks of age, which were maintained under specific pathogen-free conditions. We cultured *L. infantum* (MHOM/BR/1974/PP75), *L. amazonensis* (IFLA/BR/1967/PH-8) and *L. donovani* (LD1S/MHOM/SD/00) stationary promastigotes at 24 °C in complete Schneider’s medium (Sigma-Aldrich, St. Louis, MO, USA) supplemented with 20% (*v*/*v*) heat-inactivated fetal bovine serum (FBS; Sigma-Aldrich, St. Louis, MO, USA), 20 mM L-glutamine, 200 U/mL penicillin and 100 µg/mL streptomycin (all from Sigma-Aldrich, St. Louis, MO, USA) at pH 7.4 [34].

### 2.2. Production of ChimT and Soluble Leishmania Antigenic (SLA) Extract

The preparation, purification and quality control of recombinant ChimT have been described in detail previously [33]. Briefly, the main T cell epitopes from *Leishmania* hypothetical protein 1 (LiHyp1, XP_001468941.1), *Leishmania* hypothetical protein 2 (LiHyp2, XP_001462854.1), prohibitin (PHB, XP_001468827.1), and eukaryotic initiation factor 5a (EIF5a, XP_001466105.1) proteins were predicted by bioinformatics, and their nucleotide sequences were used to construct the gene encoding ChimT. CD4^+^ and CD8^+^ T cell epitopes were evaluated as described previously [35]. The gene encoding ChimT was synthesized commercially in the pET28a-TEV vector by GenScript^®^ (Piscataway, NJ, USA) and recombinant ChimT was expressed in *E. coli* Artic Express cells (DE3, Agilent Technologies, Santa Clara, CA, USA) with isopropyl β-D-1-thiogalactopyranoside (IPTG; Sigma-Aldrich, St. Louis, MO, USA) induction. ChimT was purified with HisTrap HP affinity chromatography on an AKTA system (GE Healthcare, Greater Milwaukee Area, WI, USA) and SuperdexTM 200 gel filtration (GE Healthcare Life Sciences, Greater Milwaukee Area, WI, USA). Polymyxin-agarose chromatography (Sigma-Aldrich, St. Louis, MO, USA) was used to remove any residual endotoxins and analysis of the recombinant protein with the Quantitative Chromogenic Limulus Amebocyte Kit (QCL-1000 model, BioWhittaker, Walkersville, MD, USA), following the manufacturer’s instructions, showed that there was less than 10 ng of lipopolysaccharides per 1 mg of protein.

Soluble *Leishmania* antigenic (SLA) extract was prepared as described previously, with a protocol involving cycles of freezing, thawing and centrifugation of late-log-phase promastigotes of *L. amazonensis* grown in liquid culture [34].

### 2.3. Infection and Immunotherapeutic Schedules

Mice (*n* = 16 per group) were infected subcutaneously in the right hind footpad with 10^7^ *L. infantum* stationary promastigotes, and 60 days after infection, they received subcutaneously in their left hind footpad one of the following schedules as shown in Table 1.

**Figure 1:**
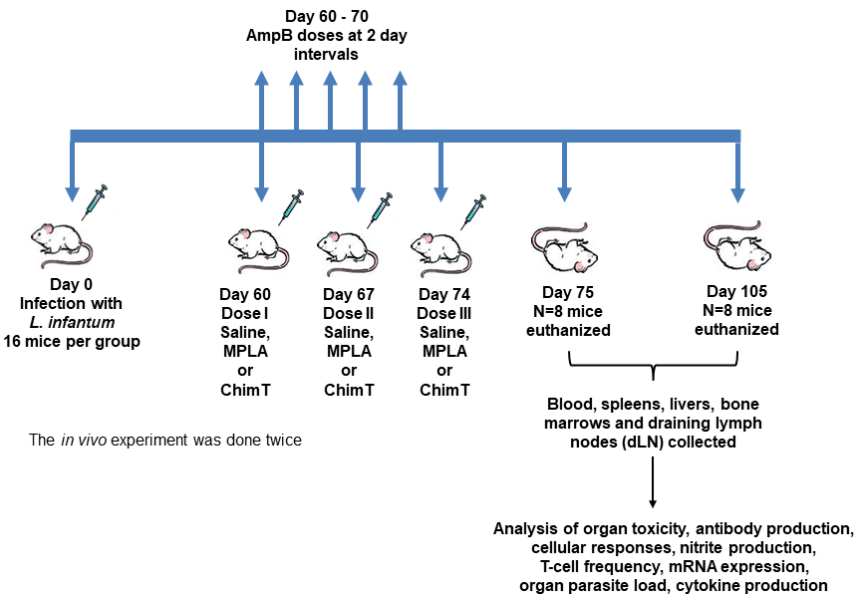
A timeline representing the infection and immunotherapeutic schedules used for this study.

Mice receiving saline, MPLA (Sigma-Aldrich, Gillingham, Dorset, UK) or ChimT were injected three times in total, subcutaneously in their left hind footpads, with a 7-day interval between each injection. Mice treated with AmpB (Sigma-Aldrich, St. Louis, MO, USA) received the drug intraperitoneally with a 2-day interval over 10 days. In all groups, mice (*n* = 8 per group, at each step) were euthanized at 1 and 30 days after the last dose. At these time points, blood samples, the spleens, livers and bone marrows (BM) and draining lymph nodes (dLN) were collected for the biochemical, parasitological and immunological assays, respectively. The infection and immunotherapeutic schedules are summarized in the graphic shown in Figure 1.

### 2.4. Organ Toxicity

Hepatic and renal toxicity was evaluated after the administration of the immunotherapeutics at both time endpoints (1 and 30 days). Blood samples were collected, and sera were separated from eight mice per group, at each step. Levels of urea and creatinine as markers of renal damage and alanine aminotransferase (ALT) and aspartate transaminase (AST) as markers of hepatic damage were quantified in the sera using commercial kits (Labtest Diagnostica^®^, Belo Horizonte, Minas Gerais, Brazil), following the manufacturer’s instructions. Sera from uninfected and untreated mice (*n* = 5, naive) were used as controls.

### 2.5. Antibody Production

The humoral response was evaluated at both endpoints after the administration of immunotherapeutics. The levels of IgG, IgG1 and IgG2a antibodies specific for ChimT and/or SLA were measured in sera collected from animals (*n* = 8 per group, at each step). The most appropriate concentration of antigen and serum dilutions to be used in the assays was determined using previously established titration curves. Microtiter plates (Jetbiofil^®^, Belo Horizonte, Minas Gerais, Brazil) were coated with ChimT or SLA (0.5 and 1.0 µg/well, respectively) in coating buffer (50 mM carbonate buffer, pH 9.6), for 16 h at 4 °C. Free binding sites were blocked using 250 µL of PBS-T (PBS plus Tween 20 0.05% *v*/*v*) with 5% (*w*/*v*) bovine serum albumin (BSA) for 1 h at 37 °C. After washing the plates five times with PBS-T, they were incubated with 100 µL of individual sera (1/100 diluted in PBS-T) for 1 h at 37 °C. Plates were washed five times in PBS-T and incubated with antibodies specific to IgG1 (rat anti-mouse IgG1 secondary antibody, peroxidase-conjugated antibody, catalog SA1-35640, Invitrogen, Waltham, MA, USA) or IgG2a (rat anti-mouse IgG2a secondary antibody, peroxidase-conjugated antibody, catalog SA1-35646, Invitrogen, Waltham, MA, USA) antibodies, used at 1/10,000 dilution in PBS-T. After incubation for 1 h at 37 °C, the wells were washed five times with PBS-T and reactions were developed using H_2_O_2_, ortho-phenylenediamine and citrate–phosphate buffer, pH 5.0, for 30 min in the dark. Reactions were stopped by adding 2N H_2_SO_4_, and optical density (OD) values were read in a spectrophotometer (Molecular Devices, Spectra Max Plus, San Jose, CA, USA) at λ492 nm [33].

### 2.6. Cellular Response and Nitrite Production

Spleens were collected from animals at both endpoints (*n* = 8 per group, in each step), and spleen cells (5 × 10^6^ per well) were plated in complete RPMI 1640 medium in duplicate in 24-well plates (Nunc), kept unstimulated (medium alone) or stimulated with ChimT or SLA (10.0 and 25.0 μg/mL, respectively), for 48 h at 37 °C in 5% (*v*/*v*) CO_2_. The levels of IFN-γ, IL-4, IL-10 and IL-12 cytokines were quantified in the collected culture supernatants with capture ELISA using commercial kits (catalogs 555138, 555232, 555252 and 555256, respectively; OptEIA TM set mouse kits; BD Pharmingen, San Diego, CA, USA), following the manufacturer’s instructions. Nitrite production in the culture supernatants was quantified by the Griess reaction. Briefly, 50 µL of culture supernatant was mixed with an equal volume of Griess reagent and absorbances were measured at λ540 nm. The concentration of nitrite was then calculated from a standard curve [33].

### 2.7. Flow Cytometry

Flow cytometry was used to evaluate the frequency of IFN-γ- and IL-10-producing polyfunctional T cells in the SLA-stimulated cell cultures. For this, spleen cells (5 × 10^6^ per well) were collected from mice at 30 days after the end of the schedules, and cells were incubated in complete RPMI medium in 96-well round-bottom plates and kept unstimulated (medium alone) or stimulated with SLA (25.0 μg/mL) for 48 h at 37 °C in 5% (*v*/*v*) CO_2_. Brefeldin A (10.0 mg/mL; Sigma-Aldrich, St. Louis, MO, USA) was added to the cultures after 4 h, and incubated for 4 h. Cells were then stained with Fixable Viability Stain 450 (FVS450, BD Biosciences, San Diego, CA, USA) for 15 min at room temperature, and labelled with antibodies against CD4^+^ (BV605 anti-mouse, clone RM4-5, catalog 563121) or CD8^+^ (BV786 anti-mouse, clone 53-6.7, catalog 563332) T cells, for 30 min at room temperature. They were then fixed with FACS fixing solution, washed and permeabilized with PBS plus 0.5% (*w*/*v*) saponin; the cells were then reacted with antibodies against IFN-γ (AF700 anti-mouse, clone XMG1.2, catalog 557998) and IL-10 (APC anti-mouse, clone JES5-16E, catalog 554468) for 30 min at room temperature. Phorbol 12-myristate 13-acetate (5.0 ng/mL; PMA) and ionomycin (1.0 mg/mL) were used as stimulus controls. All antibodies were obtained from BD Biosciences (San Diego, CA, USA). Cells were acquired (100,000 events) on a LSR Fortessa cytometer (BD Biosciences, San Diego, CA, USA) using FACSDiva software. For analysis in FlowJo software (v10.9), dead cells were excluded after FVS450 staining and viable cells were gated for stained CD4^+^ and CD8^+^ T cells. Data are expressed as indices, which were calculated by the ratio between values found in the stimulated versus unstimulated cultures. In addition, the gating strategy used to evaluate the frequency of cytokine-producing T cells is shown in Supplementary Figure S1.

### 2.8. IFN-γ mRNA Expression

RT-qPCR was used to evaluate the expression of IFN-γ mRNA in the SLA-stimulated cell cultures [36]. Mouse spleens (*n* = 8 per group) were collected at 30 days after the schedules, and RNA was extracted using TRIzol reagent (Invitrogen, Carlsbad, CA, USA), according to the manufacturer’s instructions. The material was suspended in UltraPure™ DNase/RNase-Free Distilled Water (Invitrogen, Carlsbad, CA, USA) and the RNA concentration was measured spectrophotometrically in a NanoDrop LITE reader (Thermo Scientific, Waltham, MA, USA), with absorbance ratios of λ260/280 nm. RNA quality was evaluated with electrophoresis in a 1.5% agarose gel and it was treated for 15 min at room temperature with DNAse (Invitrogen, Carlsbad, CA, USA). DNAse enzyme was deactivated with 25 mM EDTA for 10 min at 65 °C, and RNA (2 μg) was reverse transcribed using an Applied Biosystems High-Capacity cDNA Reverse Transcription Kit (Thermo Fisher, Waltham, MA, USA), forming cDNA with the following parameters: 25 °C for 10 min, 37 °C for 120 min and 85 °C for 5 min. RT-qPCR was done using Applied Biosystems PowerUp^TM^ SYBR^TM^ Green PCR master mix (Thermo Fisher, Waltham, MA, USA) and the gene-specific primers for IFN-γ of 5′-TCAAGTGGCATAGATGTGGAAGAA-3′ (Forward) and 5′-TGGCTCTGCAGGATTTTCATG-3′ (Reverse) in a 7900HT Thermocycler (Applied Biosystems, Bedford, MA, USA). Transcripts were normalized using ACTB and GAPDH housekeeping genes. The procedure was optimized by adjusting the primer concentrations to 5, 10 and 15 pmol to test for optimal specificity and efficiency. The purity of PCR products was evaluated with melting curves and gel electrophoresis. The cycle parameters were (i) initial denaturation at 95 °C for 10 min, (ii) 40 cycles at 95 °C for 15 s and (iii) annealing/extension at 60 °C for 1 min, followed by (iv) a dissociation stage for recording the melting curve. Results are shown graphically as the fold changes in gene expression by using the mean ± standard deviation of target gene. Data were analyzed according to the relative expression using the 2^−ΔΔCT^ method [37].

### 2.9. Parasite Load Quantified by Limiting Dilution Technique

The parasite load was quantified in mouse organs at both endpoints using a limiting dilution technique [35]. Briefly, the livers, spleens, BM and dLN were collected from mice (*n* = 8 per group, at each time point), and they were weighed and homogenized in a glass tissue grinder in sterile PBS. Centrifugation at 150× *g* was performed to remove debris and the cells were then concentrated by centrifugation at 2000× *g*. The pellets were suspended in complete Schneider’s medium and 220 µL of cultures was plated onto 96-well flat-bottom microtiter plates (Nunc). A serial dilution was performed in log scale using complete medium (10^−1^ to 10^−12^ dilution). Each sample was plated in triplicate and read 7 days after the beginning of the culture incubation at 24 °C. Results are expressed as the titer negative log adjusted per milligram of organ.

### 2.10. Splenic Parasitism Evaluated by qPCR

Splenic parasitism was quantified with qPCR [36]. Briefly, mouse spleens were collected at 30 days after the schedules (*n* = 8 per group), and DNA was extracted using the Wizard Genomic DNA Purification Kit (Promega Corporation, Madison, WI, USA) and suspended in UltraPure™ DNase/RNase-Free Distilled Water (Invitrogen, Carlsbad, CA, USA). The parasite load was estimated using primers to amplify *L. infantum* kDNA: 5′-CCTATTTTACACCAACCCCCAGT-3′ (Forward) and 5′-GGGTAGGGGCGTTCTGCGAAA-3′ (Reverse). Mouse β-actin gene (Forward: 5′-CAGAGCAAGAGAGGTATCC-3′ and Reverse: 5′-TCATTGTAGAAGGTGTGGTGC-3′) was used as endogenous control. Reactions were processed in 7500 HT (96-well plate; Applied Biosystems, Bedford, MA, USA) using the Applied Biosystems PowerUp^TM^ SYBR^TM^ Green PCR master mix (5 μL; Thermo Fisher, Waltham, MA, USA), added with 2 mM of each primer (1 μL) and 4 μL of DNA (25.0 ng/μL). Samples were incubated at 95 °C for 10 min and subjected to 40 cycles of 95 °C for 15 s and 60 °C for 1 min. At each time point, fluorescence data were collected, and results were calculated by interpolation from a standard curve included in the same run, which was conducted in duplicate and is expressed as the number of parasites per total DNA.

### 2.11. Treatment of Infected Macrophages and Evaluation of In Vitro Cytokine Production

The efficacy of ChimT in stimulating *Leishmania*-infected macrophages to kill parasites was examined as described previously [38]. Briefly, murine cells (5 × 10^5^) were plated onto round glass coverslips inside the wells of 24-well plates (Nunc) in RPMI 1640 medium supplemented with 20% (*v*/*v*) FBS and 2 mM L-glutamine (Sigma-Aldrich, St. Louis, MO, USA) at pH 7.4. After 24 h at 37 °C in 5% (*v*/*v*) CO_2_, stationary promastigotes were added (at a ratio of 10 parasites per macrophage) and cultures were maintained for 24 h at 37 °C in 5% (*v*/*v*) CO_2_. Next, free parasites were removed by washing with RPMI 1640 medium, and infected macrophages were incubated with ChimT (2.5, 5.0, 10.0 and 20.0 µg/mL), AmpB (0.5, 1.0, 2.0 and 5.0 µg/mL) or ChimT/MPLA/AmpB (2.5, 5.0, 10.0 and 15.0 µg/mL) for 48 h at 37 °C in 5% (*v*/*v*) CO_2_. Cells were washed in RPMI 1640 and incubated with 4% (*w*/*v*) paraformaldehyde for 15 min, and then treated with 70% (*v*/*v*) ethanol on ice for 4 h, and again washed three times with sterile PBS. Macrophages were stained with Giemsa, and the infection percentage and the number of recovered amastigotes were determined by counting 200 cells (*n* = 3 sample counts) using an optical microscope. The culture supernatants were collected and the levels of IFN-γ, IL-4, IL-10 and IL-12 cytokines were quantified with capture ELISA using commercial kits (BD OptEIA TM set mouse, Pharmingen^®^, San Diego, CA, USA), according to manufacturer’s instructions.

### 2.12. Statistical Analyses

Data were analyzed with GraphPad Prism^TM^ (version 6.0 for Windows). A one-way analysis of variance and Student’s t test were used to compare the data from the groups, with differences considered significant with *p* < 0.05. The complete in vivo experiment was performed twice, similar results were obtained, and the data shown are representative of one of the experiments.

## 3. Results

### 3.1. The Association between ChimT, MPLA and AmpB Reduces the Organic Toxicity in the Treated Animals

Initially, we examined the toxicity of our immunotherapeutic regimens in BALB/c mice, by quantifying markers of renal (urea and creatinine) and hepatic (alanine aminotransferase (ALT) and aspartate transaminase (AST)) damage, in blood samples taken at day 1 and day 30 after the end of the treatment schedules. In general, mice that received ChimT/MPLA and ChimT/MPLA/AmpB presented the lowest levels of these enzymatic markers, when compared to values found in the other groups, at both 1 and 30 days after immunotherapy (Table 2). In addition, ChimT and ChimT/AmpB presented lower levels of urea, creatinine, ALT and AST as compared to saline, AmpB and MPLA control groups; however, the enzymatic marker levels of these two groups were higher as compared to those found in sera from mice receiving ChimT/MPLA or ChimT/MPLA/AmpB.

### 3.2. Higher Production of Anti-Parasite IgG2a Antibody in Mice Treated with ChimT/MPLA/AmpB

**Figure 2:**
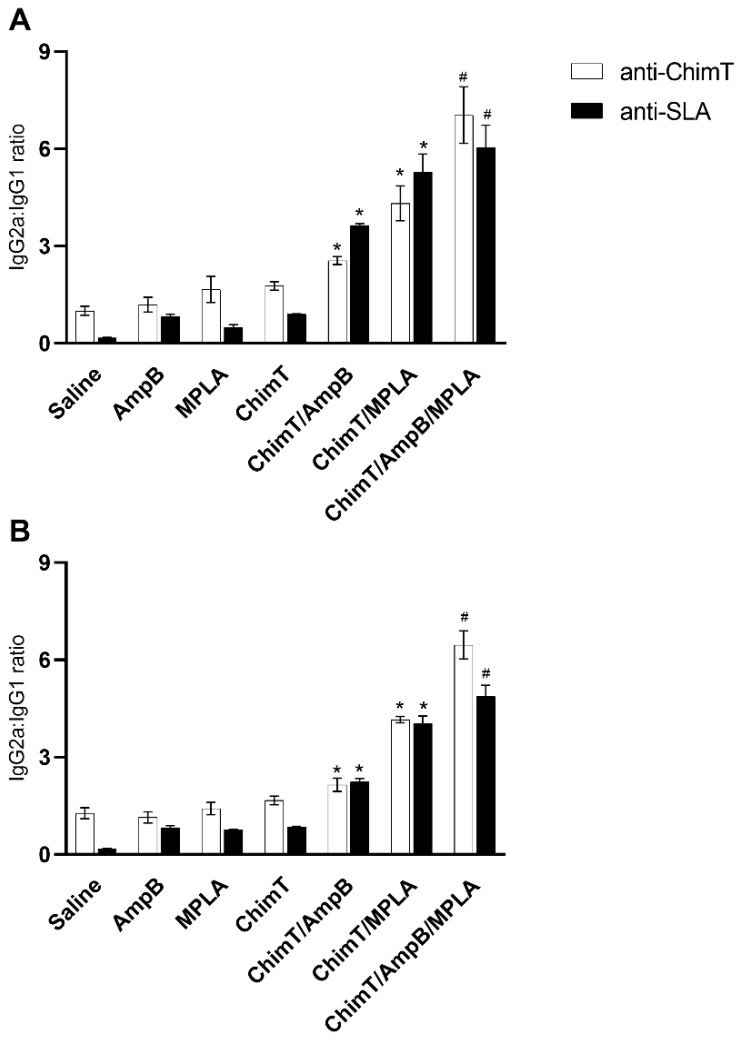
Antibody production induced after immunotherapy. The humoral response was evaluated using sera collected from animals (*n* = 8 per group at each time) at 1 and 30 days after immunotherapy. Levels of anti-ChimT protein and anti-SLA IgG1 and IgG2a isotype antibodies were evaluated with indirect ELISA. The ratios between IgG2a and IgG1 levels were calculated, and the data are shown for (**A**) day 1 and (**B**) day 30 after immunotherapy. The bars define the mean and the error bars the standard deviation of the groups. * Indicates significant statistical difference with the saline, AmpB, MPLA and ChimT groups (*p* < 0.0001). ^#^ Indicates significant statistical difference with the saline, AmpB, MPLA, ChimT, ChimT/AmpB and ChimT/MPLA groups (*p* < 0.001).

We used an indirect ELISA to evaluate the levels of anti-ChimT and anti-SLA IgG1 and IgG2a antibody responses from mice at both endpoints, i.e., at day 1 and day 30 after the end of the immunotherapy. Sera from mice in the ChimT/MPLA, ChimT/AmpB and ChimT/MPLA/AmpB groups contained significantly higher ratios of anti-ChimT and anti-parasite IgG2a:IgG1 antibodies compared to the control groups (saline, AmpB and MPLA) at both time points (Figure 2A,B). The humoral response induced by ChimT alone was not significantly different to the controls, and, in general, the control mice produced higher anti-parasite IgG1 levels, indicating a humoral response profile more akin to the development of a Th2-type response.

### 3.3. Mice Receiving ChimT/MPLA/AmpB Develop a Th1-Type Cellular Immune Response

**Figure 3:**
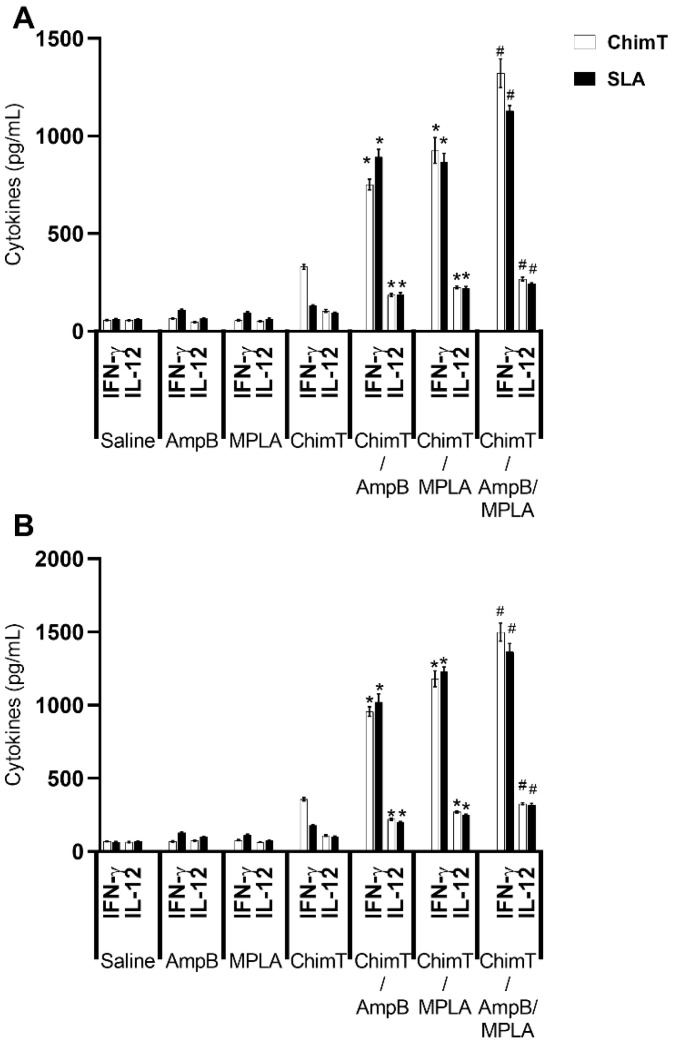
IFN-γ and IL-12 cytokine production evaluated on day 1 and day 30 after immunotherapy. Spleen cell cultures were obtained from mice (*n* = 8 per group at each time) at 1 and 30 days after immunotherapy. Cells (5 × 10^6^ per well) were stimulated with ChimT or SLA (10.0 and 25.0 µg/mL, respectively) for 48 h at 37 °C in 5% (*v*/*v*) CO_2_. The supernatant was collected and levels of IFN-γ and IL-12 were measured with capture ELISA. Bars indicate the mean and error bars indicate the standard deviation of the groups at (**A**) 1 and (**B**) 30 days after immunotherapy. * Indicates significant statistical difference with the saline, AmpB, MPLA and ChimT groups (*p* < 0.0001). ^#^ Indicates significant statistical difference with the saline, AmpB, MPLA, ChimT, ChimT/AmpB and ChimT/MPLA groups (*p* < 0.001).

**Figure 4:**
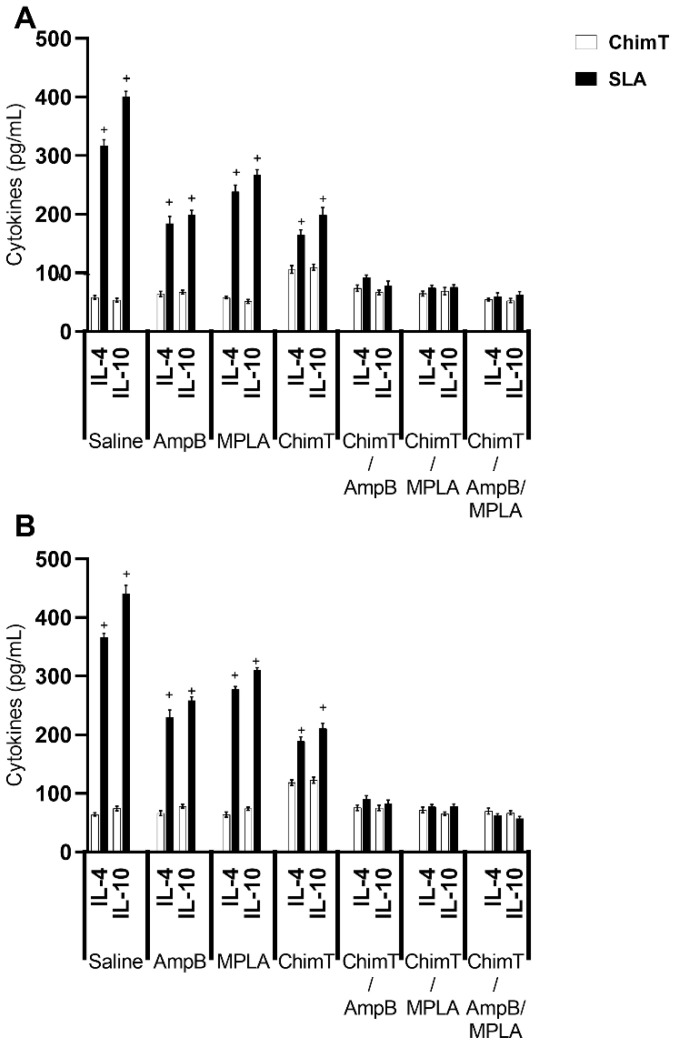
IL-4 and IL-10 cytokine production evaluated on day 1 and day 30 after immunotherapy. Spleen cell cultures were obtained from mice (*n* = 8 per group at each time) at 1 and 30 days after immunotherapy. Cells (5 × 10^6^ per well) were stimulated with ChimT or SLA (10.0 and 25.0 µg/mL, respectively) for 48 h at 37 °C in 5% (*v*/*v*) CO_2_. The supernatant was collected and levels of IL-4 and IL-10 were measured with capture ELISA. Bars indicate the mean and error bars indicate the standard deviation of the groups at (**A**) 1 and (**B**) 30 days after immunotherapy. ^+^ Indicates significant statistical difference with the ChimT/AmpB, ChimT/MPLA and ChimT/MPLA/AmpB groups (*p* < 0.0001).

The cellular response was also evaluated by examining culture supernatants stimulated with ChimT or SLA for cytokine production with capture ELISA, IFN-γ mRNA expression using RT-qPCR, cytokine-producing T cell frequency using flow cytometry and nitrite production using the Griess reaction. Mice receiving ChimT/MPLA, ChimT/AmpB and ChimT/MPLA/AmpB developed a polarized Th1-type response, which was based on the statistically higher production of ChimT protein- and parasite-specific IFN-γ and IL-12 cytokines at both 1 day and 30 days after immunotherapy, compared to controls and ChimT alone (Figure 3). This was also associated with low levels of IL-4 and IL-10 production. Conversely, control mice (saline, AmpB and MPLA) produced higher levels of anti-parasite IL-4 and IL-10 cytokines, when compared with the ChimT/MPLA-, ChimT/AmpB- and ChimT/MPLA/AmpB-treated mice, and this reflected the occurrence of a polarized Th2-type response (Figure 4).

**Figure 5:**
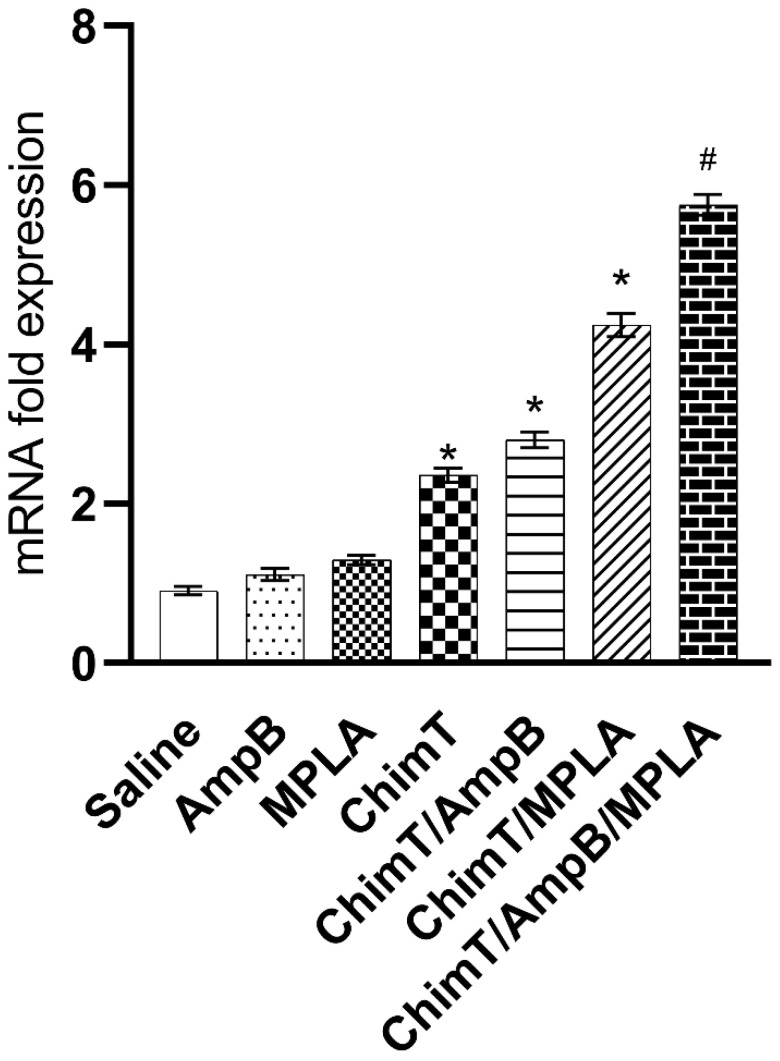
IFN-γ mRNA expression evaluated with RT-qPCR. Spleen cells were obtained from mice (*n* = 8 per group) at 30 days after immunotherapy. The cells (5 × 10^6^ per well) were stimulated with SLA (25.0 µg/mL) for 48 h at 37 °C in 5% (*v*/*v*) CO_2_ and RT-qPCR was used to evaluate the expression of IFN-γ mRNA in the stimulated cell cultures. The bars indicate the mean and the error bars indicate the standard deviation of groups. * Indicates significant statistical difference with the saline, AmpB and MPLA groups (*p* < 0.0001). ^#^ Indicates significant statistical difference with the saline, AmpB, MPLA, ChimT, ChimT/AmpB and ChimT/MPLA groups (*p* < 0.001).

Spleen cells derived from ChimT/MPLA, ChimT/AmpB and ChimT/MPLA/AmpB mice showed statistically higher levels of IFN-γ mRNA compared with the control groups (Figure 5), which is consistent with the higher production of this cytokine quantified with capture ELISA.

**Figure 6:**
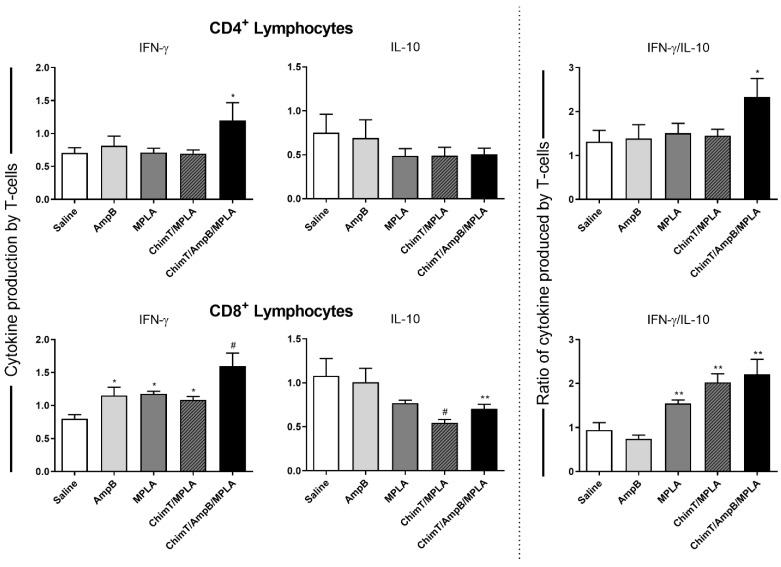
Intracytoplasmic cytokine-producing T cell frequency developed after immunotherapy. Spleen cell cultures (5 × 10^6^ per well) obtained from mice (*n* = 8 per group) 30 days after immunotherapy were stimulated with SLA (25.0 µg/mL) for 48 h at 37 °C in 5% (*v*/*v*) CO_2_. IFN-γ and IL-10-producing CD4^+^ and CD8^+^ T cell frequency was evaluated with flow cytometry. Bars represent the IFN-γ and IL-10 production by CD4^+^ and CD8^+^ T cells. The ratio between IFN-γ and IL-10 was calculated with the T cell subpopulations, and results are shown as mean ± standard deviation of the groups. * Indicates significant statistical difference in relation to the saline group (*p* < 0.05). ** Indicates significant statistical difference in relation to the saline and AmpB groups (*p* < 0.05). ^#^ Indicates significant statistical difference in relation to the AmpB and MPLA groups (*p* < 0.05).

**Figure 7:**
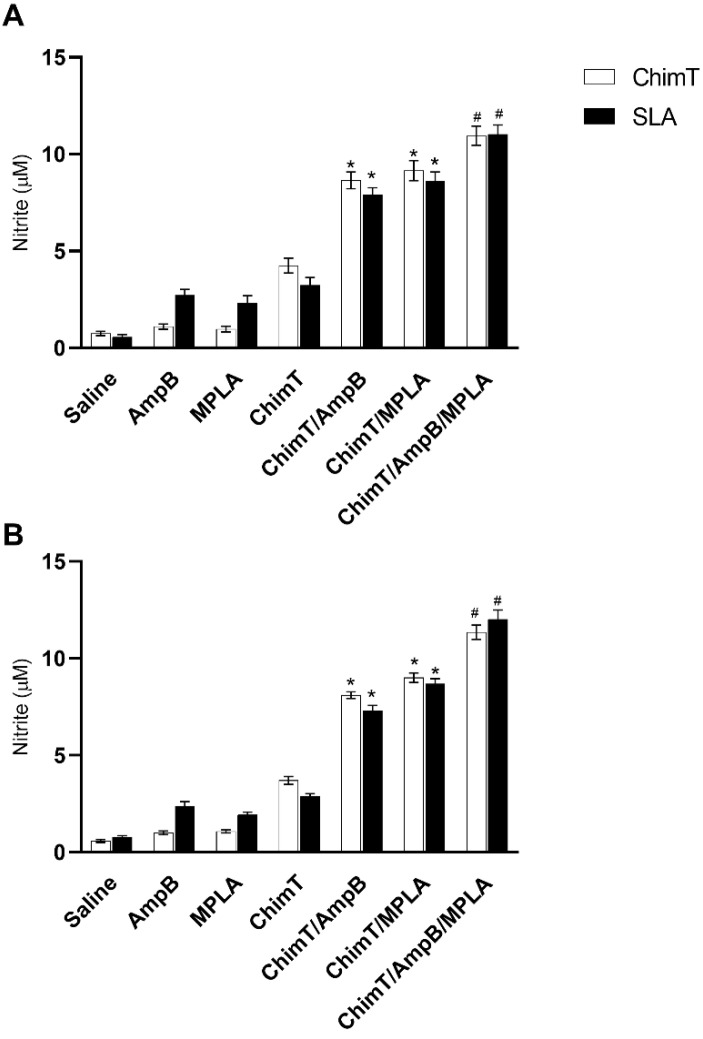
Nitrite secretion evaluated after immunotherapy. Nitrite secretion was estimated with Griess reaction in the cell culture supernatants, which were stimulated with ChimT or SLA (10.0 and 25.0 µg/mL, respectively) for 48 h at 37 °C in 5% (*v*/*v*) CO_2_. Data are shown as bars that indicate the mean with error bars denoting the standard deviation of the groups at (**A**) 1 and (**B**) 30 days after immunotherapy. * Indicates significant statistical difference with the saline, AmpB, MPLA and ChimT groups (*p* < 0.0001). ^#^ Indicates significant statistical difference with the saline, AmpB, MPLA, ChimT, ChimT/AmpB and ChimT/MPLA groups (*p* < 0.001).

A flow cytometry assay showed that mice from the ChimT/MPLA/AmpB group had a statistically higher frequency of CD4^+^ and CD8^+^ T cell subtypes producing IFN-γ, and lower levels of IL-10 production (Figure 6). Otherwise, cells from the control mice had higher frequencies of IL-10-producing T cells, but without statistical significance. Additionally, control group mice presented statistically lower IFN-γ/IL10 ratios, as compared to the others (Figure 6). Finally, spleen cell cultures from ChimT/MPLA, ChimT/AmpB and ChimT/MPLA/AmpB mice stimulated with ChimT and SLA had significantly higher levels of antileishmanial nitrite production at both day 1 (Figure 7A) and day 30 (Figure 7B), when compared to values found in the controls.

### 3.4. Treatment with ChimT/MPLA/AmpB Reduces Parasitism in Treated Mice

**Figure 8:**
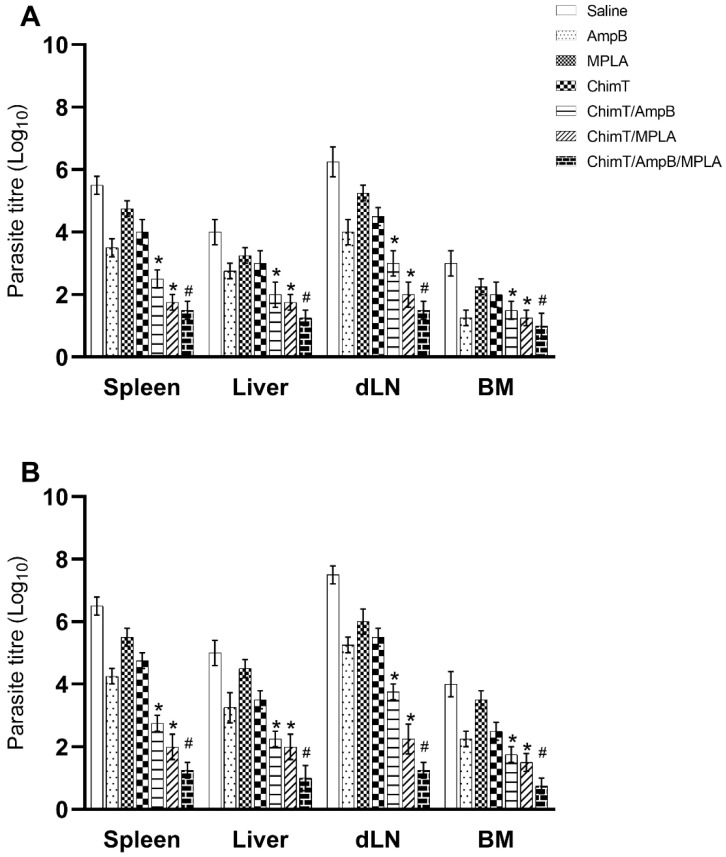
Estimation of the parasite load using a limiting dilution technique. Organ parasitism was evaluated in the animals at 1- and 30-day post schedules (*n* = 8 mice per group, at each time); their livers, spleens, draining lymph nodes (dLN) and bone marrows (BM) were collected and the parasite load was estimated with a limiting dilution technique. Data are shown as bars that indicate the mean and error bars the standard deviation of the groups at (**A**) 1 and (**B**) 30 days after immunotherapy. * Indicates significant statistical difference with the saline, AmpB, MPLA and ChimT groups (*p* < 0.0001). ^#^ Indicates significant statistical difference with the saline, AmpB, MPLA, ChimT, ChimT/AmpB and ChimT/MPLA groups (*p* < 0.001).

**Figure 9:**
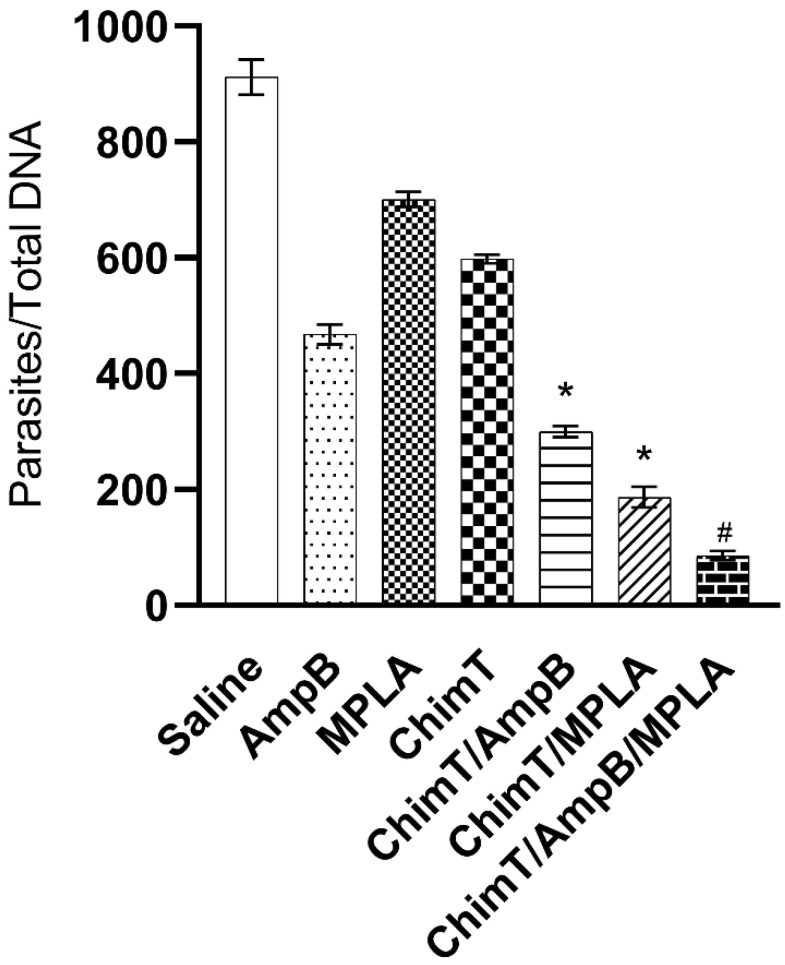
Splenic parasitism evaluated with qPCR technique. The parasite load was estimated with qPCR when mouse spleens (*n* = 8 per group) were collected 30 days after immunotherapy. Data are presented as bars indicating the mean and error bars the standard deviation of the groups. * Indicates significant statistical difference with the saline, AmpB, MPLA and ChimT groups (*p* < 0.0001). ^#^ Indicates significant statistical difference with the saline, AmpB, MPLA, ChimT, ChimT/AmpB and ChimT/MPLA groups (*p* < 0.001).

We evaluated parasitism at day 1 (Figure 8A) and day 30 (Figure 8B) after immunotherapy, and the data shows that ChimT/MPLA, ChimT/AmpB and ChimT/MPLA/AmpB induced significant reductions in the parasite load in the livers, spleens, BM and dLN of the treated and infected animals, as compared to values found in the controls. We also investigated splenic parasitism with qPCR at 30 days after therapy, and the qPCR data confirmed the measured parasite load data, whereby significant reductions in splenic parasitism were found in the ChimT/MPLA, ChimT/AmpB and ChimT/MPLA/AmpB mice, compared to the controls (Figure 9). Notably, the association of ChimT, MPLA adjuvant and AmpB induced marginally higher reductions in organ parasitism, when compared to the association of ChimT with MPLA or with AmpB.

### 3.5. ChimT Stimulates In Vitro Infected Macrophages to Kill Parasites and to Produce Th1-Type Cytokines

We examined the in vitro efficacy of the ChimT/MPLA/AmpB mixture for stimulating murine macrophages infected with *L. amazonensis*, *L. donovani* and *L. infantum* promastigotes, using AmpB as a reference antileishmanial drug. The percentage of untreated, control macrophages infected with *L. amazonensis*, *L. donovani* and *L. infantum* promastigotes was 97.6%, 96.3% and 95.2%, respectively (Table 3). There was a dose-dependent reduction in the percentage of infected macrophages after treatment and incubation with the highest dose of ChimT/MPLA/AmpB tested (15.0 µg/mL) resulted in 9.8%, 8.6% and 4.5% of macrophages infected with *L. amazonensis*, *L. donovani* and *L. infantum*, respectively (Table 3). These data are significantly better than those obtained when ChimT or AmpB were used at their highest dose. The number of *L. amazonensis*, *L. donovani* and *L. infantum* amastigotes recovered from untreated macrophages was 8.7, 7.9 and 8.3, respectively (Table 3). When incubated with ChimT/MPLA/AmpB, these values fell to 0.2, 0.3 and 0.2 parasite, which are marginally superior to the values obtained with ChimT or AmpB.

We also measured the levels of IFN-γ, IL-4, IL-10 and IL-12 cytokines in the culture supernatants from treated macrophages, and significantly higher and dose-dependent increases in IFN-γ and IL-12 production were found with all three parasite species, which was associated with lower levels of IL-4 and IL-10 (Table 4). Moreover, in most cases, significantly higher production of the Th1-type cytokines (IFN-γ and IL-12) was found in the culture supernatants after incubation with ChimT/MPLA/AmpB across the range of doses tested.

## 4. Discussion

Multi-antigenic T cell epitope-based vaccines are considered as good candidates for VL prophylaxis as they contain distinct immunogenic parts derived from several different parasite proteins, which have already been shown to be immunogenic and/or protective against infection [25,26,39]. In addition, associating vaccines with antileishmanial drugs in an immunotherapy protocol could reduce infection, based on the rationale that the drug eliminates the parasite directly and that the vaccine, with or without an adjuvant, stimulates the host immune response [40,41]. In the present study, we used this dual therapeutic approach with ChimT chimera vaccine, adjuvanted with MPLA and associated with AmpB, to treat mice that had been infected with *L. infantum* parasites. The key findings from our study are that treatment with ChimT plus MPLA and AmpB (i) was not significantly toxic, (ii) induced the development of a Th1-type cellular and humoral immune response and (iii) significantly reduced organ parasitism. Moreover, ChimT protein significantly stimulated in vitro *Leishmania*-infected macrophages to kill parasites and to produce Th1-type cytokines (IFN-γ and IL-12). The combination of the vaccine–adjuvant and drug could be considered for future studies to develop an immunotherapeutic for use in humans with VL.

In our study, mice receiving saline, MPLA or AmpB alone developed a polarized Th2-type response, which was reflected in a higher parasite load and marginally increased organic toxicity. Conversely, mice receiving ChimT/MPLA, ChimT/AmpB and mainly ChimT/MPLA/AmpB showed an up-regulation of IFN-γ and IL-12 cytokine production, which was associated with a decreased presence of Th2-type cytokines and reflected the induction of protective immunity against the disease. The dominant effect of a Th1-type response in these animals is the activation of microbicidal and effector killing mechanisms by the infected cells, which was reflected in the higher secretion of antileishmanial nitrite as a proxy for nitric oxide, which contributed to clearing *Leishmania* [42]. In addition, in vitro experiments using three *Leishmania* spp. showed that ChimT protein was immunogenic towards infected murine macrophages, suggesting that this protein could be a potential immunotherapy for treating infection and infected cells [43]. Similar immune profiles have been reported by other studies with other vaccine [44,45] or therapeutic [46,47] candidates. We conclude that the association of ChimT with the adjuvant MPLA and drug AmpB appeared to enhance the in vivo immunotherapeutic potential of ChimT to stimulate the Th1-biased immune response and optimize the AmpB-induced therapeutic regimen by reducing its toxicity to some degree. The dual mechanism of protection involves the direct effect of the drug to reduce parasitism and of the vaccine to stimulate a clearance immune response.

The major positive outcome expected of a successful vaccine for VL would be reduction in organ parasitism, and this is a parameter to evaluate in pre-clinical studies [48,49]. Usually, the spleen and liver are the organs affected most by the parasites, which can also reach the bone marrow, draining lymph nodes and some other internal organs [50]. In the first weeks after infection is established in mammals, the amastigote develops and persists in the liver and later on in the spleen, and consequently, parasite reduction in these organs is an important parameter to be met by a successful therapy [51]. The ChimT/MPLA/AmpB combination in our study reduced splenic and hepatic parasitism. Notably, the inability of AmpB, ChimT or MPLA alone to control infection in our experimental model suggests that their association provides a synergistic antileishmanial activity. However, the immunotherapy did not completely eliminate parasites from the internal organs and suggests that higher doses and/or longer treatment periods may be needed to improve *Leishmania* clearance. Additional studies are required to test this hypothesis.

In our study, the reference drug AmpB promoted a reduction in organ parasitism but with increased organic toxicity as demonstrated by marginally higher levels of urea, creatinine, ALT and AST enzymes. The drug alone appears to act directly on the parasites, with the induction of a low-level Th1-type response, whereas in association with other antileishmanial candidates, such as 8-hydroxyquinoline [52], dextrin [10,11], lupeol [53] and 6-gingerol [54], it exerts a synergistic effect and participates in the development of a polarized immune response and treatment against *Leishmania* infection. Importantly, the ChimT/MPLA/AmpB immunotherapy did not increase overall toxicity and suggests that the chimeric protein and/or immune adjuvant potentially provide some protection against the toxicity of AmpB. If the presence of these components helps to ameliorate the toxicity of AmpB, or indeed any other antileishmanial drugs, this would be an important attribute for improving uptake of human and canine immunotherapies. The absence of clinical signs and the significant decrease in the parasite burden with the ChimT/MPLA/AmpB immunotherapy protocol probably correlates with the restoration and activation of an effective immune response of the animals. Future studies could investigate the association of ChimT and the adjuvant with other antileishmanial drugs to examine if the synergy is broad or restricted.

A final important finding from our study that deserves discussion is the fact that the ChimT vaccine alone can stimulate murine macrophages to kill internalized parasites, of all three different *Leishmania* spp., and to produce cytokines. ChimT was effective at activating *L. infantum* parasite-infected cells, thereby reducing parasite loads and inducing high levels of IFN-γ and IL-12, and low levels of IL-4 and IL-10. Thus, post-infection treatment with the vaccine suggests that the immune cells were activated to produce a Th1-type response that killed the parasites, possibly via a nitric oxide-dependent mechanism. The hypothesis that ChimT is active against other *Leishmania* species should be tested in future in vivo studies, including larger animal models such as the hamster and dog. Additionally, new assays should be performed for longer periods of time than 30 days after the immunotherapy, to define a possible long-term therapeutic efficacy as well as to evaluate a sterile antileishmanial cure. The limitations of our study include the lack of a more detailed analysis of vaccine–drug toxicity and more refined analysis of vaccine–drug combinations, aiming to reduce the number of doses and/or their concentrations.

## 5. Conclusions

There is an urgent need for effective immunotherapies for human and canine VL. The data presented here should be considered as a proof-of-concept that the combination of ChimT/MPLA/AmpB deserves further development as an immunotherapy against VL.

## Figures and Tables

**Table 1:** Immunotherapeutic schedules for mice experimentally infected with *L. infantum*.

Treatment	Dose/Mouse
Saline	50 µL/mouse
AmpB	1 mg/kg/weight dose/mouse
MPLA	20 µg dose/mouse
ChimT	20 µg per dose/mouse
ChimT/AmpB	20 µg ChimT and 1 mg/kg/weight AmpB dose/mouse
ChimT/MPLA	20 µg ChimT and 20 µg MPLA dose/mouse
ChimT/MPLA/AmpB	20 µg ChimT, 20 µg MPLA, 1 mg/kg/weight AmpB dose/mouse

**Table 2:** Evaluation of in vivo toxicity. Organ toxicity was evaluated in the infected animals, at 1 and 30 days after the application of distinct immunotherapeutic schedules, by measuring the levels of urea and creatinine as renal damage markers and alanine aminotransferase (ALT) and aspartate aminotransferase (AST) as hepatic damage markers. Serum samples collected from non-infected and non-treated (naive) mice were used as negative controls. Data are shown as the means with standard deviations and were analyzed with a paired *t* test, with raw statistical data for the comparative groups presented in Supplementary Table S1.

**Urea**					
	**1 Day**		**30 Days**		
**Groups**	**Mean**	**Std. Deviation**	**Mean**	**Std. Deviation**	**Paired *t* Test**
Naive	13.3	1.7	19.5	1.3	0.8729
Saline	24.0	1.8	26.8	1.7	0.1627
AmpB	28.8	3.8	31.0	1.8	0.1697
MPLA	22.3	1.7	24.5	1.3	0.1697
ChimT	19.8	2.6	19.5	1.3	0.8543
ChimT/AmpB	20.5	1.3	17.8	1.3	0.0222
ChimT/MPLA	16.3	2.2	15.5	1.3	0.6807
ChimT/AmpB/ MPLA	17.3	1.7	14.5	1.8	0.1152
Creatinine					
	1 day		30 days		
Groups	Mean	Std. Deviation	Mean	Std. Deviation	Paired *t* test
Naive	1.1	0.2	2.0	0.1	0.4228
Saline	2.9	0.3	2.9	0.2	0.3910
AmpB	3.2	0.4	3.3	0.3	0.4740
MPLA	2.6	0.2	2.6	0.2	0.2152
ChimT	1.7	0.2	2.0	0.1	0.1817
ChimT/AmpB	1.8	0.2	1.8	0.2	0.8088
ChimT/MPLA	1.5	0.3	1.5	0.2	0.3910
ChimT/AmpB/ MPLA	1.6	0.3	1.4	0.3	0.3542
Alanine aminotransferase (ALT)					
	1 day		30 days		
Groups	Mean	Std. Deviation	Mean	Std. Deviation	Paired *t* test
Naive	9.0	0.8	9.8	1.0	0.3910
Saline	26.0	1.8	29.0	2.2	0.0577
AmpB	30.5	1.3	31.3	3.3	0.6714
MPLA	23.0	1.8	25.3	1.3	0.2007
ChimT	20.0	2.2	23.3	1.7	0.1438
ChimT/AmpB	21.5	2.7	19.5	1.3	0.3081
ChimT/MPLA	12.5	1.3	15.5	1.3	0.0805
ChimT/AmpB/ MPLA	13.0	2.2	14.0	1.8	0.5943
Aspartate transaminase (ALT)					
	1 day		30 days		
Groups	Mean	Std. Deviation	Mean	Std. Deviation	Paired *t* test
Naive	11.3	2.2	10.8	1.7	0.6042
Saline	24.8	1.8	24.8	1.9	0.3910
AmpB	28.0	2.2	29.3	1.7	0.1411
MPLA	21.8	2.6	21.0	1.8	0.7177
ChimT	18.5	2.1	18.0	1.9	0.6376
ChimT/AmpB	19.0	1.9	19.0	2.2	1.0000
ChimT/MPLA	14.3	1.3	15.0	1.8	0.3910
ChimT/AmpB/ MPLA	15.0	1.7	13.8	2.5	0.5367

**Table 3:** Treatment of the *Leishmania*-infected macrophages. Murine cells were infected with *L. amazonensis, L. donovani* or *L. infantum* and later incubated with ChimT (2.5, 5.0, 10.0 and 20.0 µg/mL), amphotericin B (0.5, 1.0, 2.0 and 5.0 µg/mL) or ChimT/MPLA/AmpB (2.5, 5.0, 10.0 and 15.0 µg/mL). The percentage of infected macrophages and the number of recovered amastigotes were determined by counting 200 cells in triplicate. Control group indicates untreated and infected macrophages. Results are shown as mean ± standard deviation of the groups.

**Products**	**Concentration (µg/mL)**	**Percentage of Infected Macrophages after Treatment**		
		***L. amazonensis***	***L. donovani***	***L. infantum***
ChimT	20.0	24.5 ± 2.7	19.8 ± 3.3	13.2 ± 1.7
	10.0	40.5 ± 3.3	35.4 ± 4.3	26.7 ± 2.9
	5.0	53.4 ± 2.8	49.8 ± 2.8	42.3 ± 3.3
	2.5	70.5 ± 3.7	65.5 ± 3.7	59.8 ± 4.4
Amphotericin B	5.0	19.8 ± 3.0	16.7 ± 2.0	10.4 ± 1.0
	2.0	35.4 ± 2.8	28.7 ± 2.5	21.3 ± 1.8
	1.0	48.7 ± 3.6	40.8 ± 4.1	37.8 ± 4.1
	0.5	68.7 ± 5.0	58.7 ± 3.8	54.3 ± 2.7
ChimT/MPLA/AmpB	15.0	9.8 ± 3.3	8.6 ± 2.3	4.5 ± 1.8
	10.0	21.3 ± 4.4	16.5 ± 3.4	10.8 ± 2.6
	5.0	36.5 ± 6.2	28.7 ± 6.3	21.4 ± 2.7
	2.5	55.6 ± 5.7	43.4 ± 5.0	38.7 ± 4.8
Control	(-)	97.6 ± 4.4	96.3 ± 3.6	95.2 ± 5.0
**Products**	**Concentration (µg/mL)**	**Number of recovered amastigotes**		
		***L. amazonensis***	***L. donovani***	***L. infantum***
ChimT	20.0	2.0 ± 0.8	1.6 ± 0.7	1.2 ± 0.6
	10.0	3.1 ± 1.2	2.8 ± 0.6	2.2 ± 0.7
	5.0	4.6 ± 1.1	4.1 ± 0.8	3.8 ± 0.9
	2.5	6.7 ± 0.9	6.0 ± 1.1	5.6 ± 1.4
Amphotericin B	5.0	1.5 ± 0.5	1.0 ± 0.3	0.9 ± 0.3
	2.0	2.4 ± 0.7	1.9 ± 0.6	1.9 ± 0.6
	1.0	3.9 ± 0.8	2.9 ± 0.8	3.1 ± 0.9
	0.5	5.9 ± 1.4	3.8 ± 0.8	5.0 ± 1.1
ChimT/MPLA/AmpB	15.0	0.2 ± 0.1	0.3 ± 0.1	0.2 ± 0.1
	10.0	0.7 ± 0.2	0.6 ± 0.3	0.8 ± 0.3
	5.0	1.9 ± 0.5	1.5 ± 0.4	1.8 ± 0.5
	2.5	4.1 ± 0.5	2.9 ± 0.6	3.6 ± 0.7
Control	(-)	8.7 ± 0.9	7.9 ± 1.2	8.3 ± 1.7

**Table 4:** Cytokine levels found in the culture supernatant from treated and infected macrophages. Murine cells were infected with *L. amazonensis*, *L. donovani* or *L. infantum* promastigotes and later maintained without incubation or incubated with ChimT (2.5, 5.0, 10.0 and 20.0 µg/mL), amphotericin B (0.5, 1.0, 2.0 and 5.0 µg/mL) or ChimT/MPLA/AmpB (2.5, 5.0, 10.0 and 15.0 µg/mL) for 48 h at 37 °C in 5% CO_2_. The culture supernatant was collected and IFN-γ, IL-4, IL-10 and IL-12 levels were measured with capture ELISA using commercial kits. Control group indicates untreated and infected macrophages. Results are shown as mean ± standard deviation of the groups.

	**Cytokine Levels in the Culture Supernatant from Treated and** ***L. amazonensis*-Infected Macrophages (pg/mL)**				
Products	Concentration (µg/mL)	IFN-γ	IL-4	IL-10	IL-12
ChimT	20.0	231.5 ± 9.8	51.2 ± 2.6	50.6 ± 5.6	149.6 ± 3.4
	10.0	152.6 ± 7.6	48.3 ± 4.0	42.3 ± 2.7	100.2 ± 3.6
	5.0	99.7 ± 4.0	41.2 ± 2.7	39.8 ± 3.0	74.5 ± 3.6
	2.5	56.5 ± 2.6	31.3 ± 3.5	35.4 ± 3.7	44.7 ± 4.0
Amphotericin B	5.0	53.3 ± 4.6	48.7 ± 5.4	51.2 ± 2.7	56.7 ± 3.3
	2.0	45.6 ± 3.4	41.2 ± 3.0	43.3 ± 5.4	50.7 ± 3.6
	1.0	41.2 ± 2.6	32.4 ± 2.7	37.6 ± 3.6	42.2 ± 3.9
	0.5	34.5 ± 2.2	29.4 ± 3.3	32.2 ± 2.0	35.3 ± 2.4
ChimT/MPLA/AmpB	15.0	405.4 ± 17.6	48.7 ± 3.8	45.4 ± 3.8	174.3 ± 2.5
	10.0	265.3 ± 13.9	40.2 ± 3.5	40.2 ± 4.6	143.4 ± 8.7
	5.0	189.8 ± 11.2	34.4 ± 2.7	31.4 ± 3.5	111.5 ± 7.6
	2.5	98.5 ± 6.7	26.4 ± 1.5	26.5 ± 1.7	76.6 ± 4.7
Control	(-)	32.4 ± 2.3	25.4 ± 1.6	30.2 ± 3.3	31.2 ± 4.1
	**Cytokine Levels in the Culture Supernatant from Treated and** ***L. donovani*-Infected Macrophages (pg/mL)**				
Products	Concentration (µg/mL)	IFN-γ	IL-4	IL-10	IL-12
ChimT	20.0	197.8 ± 7.8	50.8 ± 1.8	58.7 ± 3.7	134.5 ± 4.6
	10.0	143.2 ± 8.0	45.6 ± 3.7	51.1 ± 3.0	97.6 ± 5.0
	5.0	87.8 ± 3.7	39.8 ± 3.0	44.5 ± 2.8	68.7 ± 4.5
	2.5	49.8 ± 2.0	31.3 ± 3.5	36.5 ± 2.6	40.9 ± 3.5
Amphotericin B	5.0	58.7 ± 2.7	63.3 ± 3.8	59.8 ± 3.0	60.9 ± 4.3
	2.0	49.8 ± 4.4	59.7 ± 2.7	51.6 ± 3.6	51.7 ± 4.6
	1.0	46.5 ± 3.0	50.6 ± 3.3	45.5 ± 4.0	41.4 ± 2.9
	0.5	32.3 ± 3.1	41.2 ± 2.6	38.7 ± 2.7	34.5 ± 2.7
ChimT/MPLA/AmpB	15.0	28.7 ± 10.9	49.8 ± 5.3	49.8 ± 4.5	198.7 ± 12.1
	10.0	198.4 ± 16.7	47.6 ± 2.7	41.2 ± 3.5	156.4 ± 8.7
	5.0	132.2 ± 11.7	40.5 ± 4.3	32.2 ± 4.0	98.8 ± 3.5
	2.5	78.7 ± 4.5	34.3 ± 2.5	25.6 ± 2.7	65.5 ± 4.5
Control	(-)	24.5 ± 1.6	31.2 ± 2.5	35.6 ± 2.8	26.5 ± 1.7
	**Cytokine Levels in the Culture Supernatant from Treated and** ***L. infantum*-Infected Macrophages (pg/mL)**				
Products	Concentration (µg/mL)	IFN-γ	IL-4	IL-10	IL-12
ChimT	20.0	268.7 ± 10.4	60.5 ± 4.5	62.3 ± 2.7	157.7 ± 8.9
	10.0	177.6 ± 7.8	53.4 ± 2.6	54.4 ± 2.7	115.3 ± 5.8
	5.0	100.2 ± 6.5	45.6 ± 4.0	49.8 ± 3.6	76.6 ± 5.6
	2.5	65.5 ± 4.6	37.6 ± 4.4	40.5 ± 3.3	57.7 ± 3.5
Amphotericin B	5.0	56.5 ± 3.6	58.7 ± 3.7	58.7 ± 4.7	63.2 ± 4.9
	2.0	49.8 ± 4.4	52.3 ± 3.5	53.2 ± 2.6	57.6 ± 2.8
	1.0	40.7 ± 3.5	44.3 ± 3.0	45.6 ± 2.6	50.4 ± 3.7
	0.5	31.3 ± 2.7	39.8 ± 2.6	39.8 ± 3.4	44.3 ± 3.6
ChimT/MPLA/AmpB	15.0	365.6 ± 21.1	43.4 ± 2.7	50.4 ± 3.9	232.8 ± 15.4
	10.0	243.3 ± 16.5	38.7 ± 4.3	45.4 ± 3.5	189.8 ± 13.3
	5.0	167.6 ± 12.2	30.2 ± 3.1	38.7 ± 3.0	114.4 ± 10.9
	2.5	98.8 ± 4.5	23.2 ± 2.6	31.1 ± 2.9	78.7 ± 3.4
Control	(-)	25.5 ± 2.5	33.2 ± 2.6	35.7 ± 2.8	40.5 ± 3.3

## Data Availability

All available data are presented within the article.

## Supplementary Materials

The following supporting information can be downloaded at: https://www.mdpi.com/article/10.3390/biology12060851/s1, Figure S1: Representative plots of the gating strategy to evaluate the frequency of IFN-γ- and IL-10-producing T cells (in percentage) are shown through the Boolean gate strategy; Table S1: Raw statistical data supporting Table 1 toxicity data showing the comparisons between groups.
