# Supplementary material for: Efficacy of an Immunotherapy Combining Immunogenic Chimeric Protein Plus Adjuvant and Amphotericin B against Murine Visceral Leishmaniasis
Source: Biology (Basel). 2023 Jun 13;12(6):851. doi: 10.3390/biology12060851 (PMC10295016; doi:10.3390/biology12060851)

Supplementary material.

Supplementary Table S1. Raw statistical data supporting Table 1 toxicity data showing the comparisons between groups.

**ALT_1 Day**					**ALT_30 Day**				
**Tukey's multiple comparisons test**	**Mean Diff.**	**95.00% CI of diff.**	**Summary**	**Adjusted P Value**	**Tukey's multiple comparisons test**	**Mean Diff.**	**95.00% CI of diff.**	**Summary**	**Adjusted P Value**
Naive vs. Saline	-17	-19.88 to -14.12	****	<0.0001	Naive vs. Saline	-17	-19.88 to -14.12	****	<0.0001
Naive vs. AmpB	-20.75	-23.63 to -17.87	****	<0.0001	Naive vs. AmpB	-20.75	-23.63 to -17.87	****	<0.0001
Naive vs. MPLA	-13.25	-16.13 to -10.37	****	<0.0001	Naive vs. MPLA	-13.25	-16.13 to -10.37	****	<0.0001
Naive vs. LiHyp1	-9.75	-12.63 to -6.872	****	<0.0001	Naive vs. LiHyp1	-9.75	-12.63 to -6.872	****	<0.0001
Naive vs. LiHyp1/AmpB	-12.75	-15.63 to -9.872	****	<0.0001	Naive vs. LiHyp1/AmpB	-12.75	-15.63 to -9.872	****	<0.0001
Naive vs. LiHyp1/MPLA	-5.75	-8.628 to -2.872	****	<0.0001	Naive vs. LiHyp1/MPLA	-5.75	-8.628 to -2.872	****	<0.0001
Naive vs. LiHyp1/AmpB/MPLA	-2	-4.878 to 0.8781	ns	0.3328	Naive vs. LiHyp1/AmpB/MPLA	-2	-4.878 to 0.8781	ns	0.3328

**AST_1 Day**					**AST_30 Day**				
**Tukey's multiple comparisons test**	**Mean Diff.**	**95.00% CI of diff.**	**Summary**	**Adjusted P Value**	**Tukey's multiple comparisons test**	**Mean Diff.**	**95.00% CI of diff.**	**Summary**	**Adjusted P Value**
Naive vs. Saline	-15.5	-18.58 to -12.42	****	<0.0001	Naive vs. Saline	-15.5	-18.58 to -12.42	****	<0.0001
Naive vs. AmpB	-19.5	-22.58 to -16.42	****	<0.0001	Naive vs. AmpB	-19.5	-22.58 to -16.42	****	<0.0001
Naive vs. MPLA	-10.5	-13.58 to -7.420	****	<0.0001	Naive vs. MPLA	-10.5	-13.58 to -7.420	****	<0.0001
Naive vs. LiHyp1	-8.25	-11.33 to -5.170	****	<0.0001	Naive vs. LiHyp1	-8.25	-11.33 to -5.170	****	<0.0001

Naive vs. LiHyp1/AmpB	-10.5	-13.58 to -7.420	****	<0.0001	Naive vs. LiHyp1/AmpB	-10.5	-13.58 to -7.420	****	<0.0001
Naive vs. LiHyp1/MPLA	-4.75	-7.830 to -1.670	***	0.0007	Naive vs. LiHyp1/MPLA	-4.75	-7.830 to -1.670	***	0.0007
Naive vs. LiHyp1/AmpB/MPLA	-2	-5.080 to 1.080	ns	0.4133	Naive vs. LiHyp1/AmpB/MPLA	-2	-5.080 to 1.080	ns	0.4133

**UREA_1 Day**					**UREA_30 Day**				
**Tukey's multiple comparisons test**	**Mean Diff.**	**95.00% CI of diff.**	**Summary**	**Adjusted P Value**	**Tukey's multiple comparisons test**	**Mean Diff.**	**95.00% CI of diff.**	**Summary**	**Adjusted P Value**
Naive vs. Saline	-11.75	-15.44 to -8.063	****	<0.0001	Naive vs. Saline	-15.5	-18.93 to -12.07	****	<0.0001
Naive vs. AmpB	-15.25	-18.94 to -11.56	****	<0.0001	Naive vs. AmpB	-19.5	-22.93 to -16.07	****	<0.0001
Naive vs. MPLA	-8.75	-12.44 to -5.063	****	<0.0001	Naive vs. MPLA	-11.5	-14.93 to -8.069	****	<0.0001
Naive vs. LiHyp1	-6.25	-9.937 to -2.563	***	0.0002	Naive vs. LiHyp1	-8.25	-11.68 to -4.819	****	<0.0001
Naive vs. LiHyp1/AmpB	-7.5	-11.19 to -3.813	****	<0.0001	Naive vs. LiHyp1/AmpB	-10	-13.43 to -6.569	****	<0.0001
Naive vs. LiHyp1/MPLA	-3.75	-7.437 to -0.06263	*	0.0443	Naive vs. LiHyp1/MPLA	-5.5	-8.931 to -2.069	***	0.0004
Naive vs. LiHyp1/AmpB/MPLA	-1.75	-5.437 to 1.937	ns	0.7616	Naive vs. LiHyp1/AmpB/MPLA	-1.25	-4.681 to 2.181	ns	0.9221

**CREATININE_1 Day**					**CREATININE_30 Day**				
**Tukey's multiple comparisons test**	**Mean Diff.**	**95.00% CI of diff.**	**Summary**	**Adjusted P Value**	**Tukey's multiple comparisons test**	**Mean Diff.**	**95.00% CI of diff.**	**Summary**	**Adjusted P Value**
Naive vs. Saline	-1.7	-2.092 to -1.308	****	<0.0001	Naive vs. Saline	-2.025	-2.375 to -1.675	****	<0.0001
Naive vs. AmpB	-2.075	-2.467 to -1.683	****	<0.0001	Naive vs. AmpB	-2.275	-2.625 to -1.925	****	<0.0001

Naive vs. MPLA	-1.275	-1.667 to -0.8830	****	<0.0001	Naive vs. MPLA	-1.5	-1.850 to -1.150	****	<0.0001
Naive vs. LiHyp1	-0.85	-1.242 to -0.4580	****	<0.0001	Naive vs. LiHyp1	-1.05	-1.400 to -0.7003	****	<0.0001
Naive vs. LiHyp1/AmpB	-1.15	-1.542 to -0.7580	****	<0.0001	Naive vs. LiHyp1/AmpB	-1.15	-1.500 to -0.8003	****	<0.0001
Naive vs. LiHyp1/MPLA	-0.6	-0.9920 to -0.2080	***	0.0008	Naive vs. LiHyp1/MPLA	-0.55	-0.8997 to -0.2003	***	0.0006
Naive vs. LiHyp1/AmpB/MPLA	-0.375	-0.7670 to 0.01702	ns	0.0679	Naive vs. LiHyp1/AmpB/MPLA	-0.25	-0.5997 to 0.09965	ns	0.3004

Supplementary Figure S1. Representative plots of the gating strategy to evaluate the frequency of IFN-γ and IL-10-producing T cells (in percentage) are shown through the Boolean gate strategy.

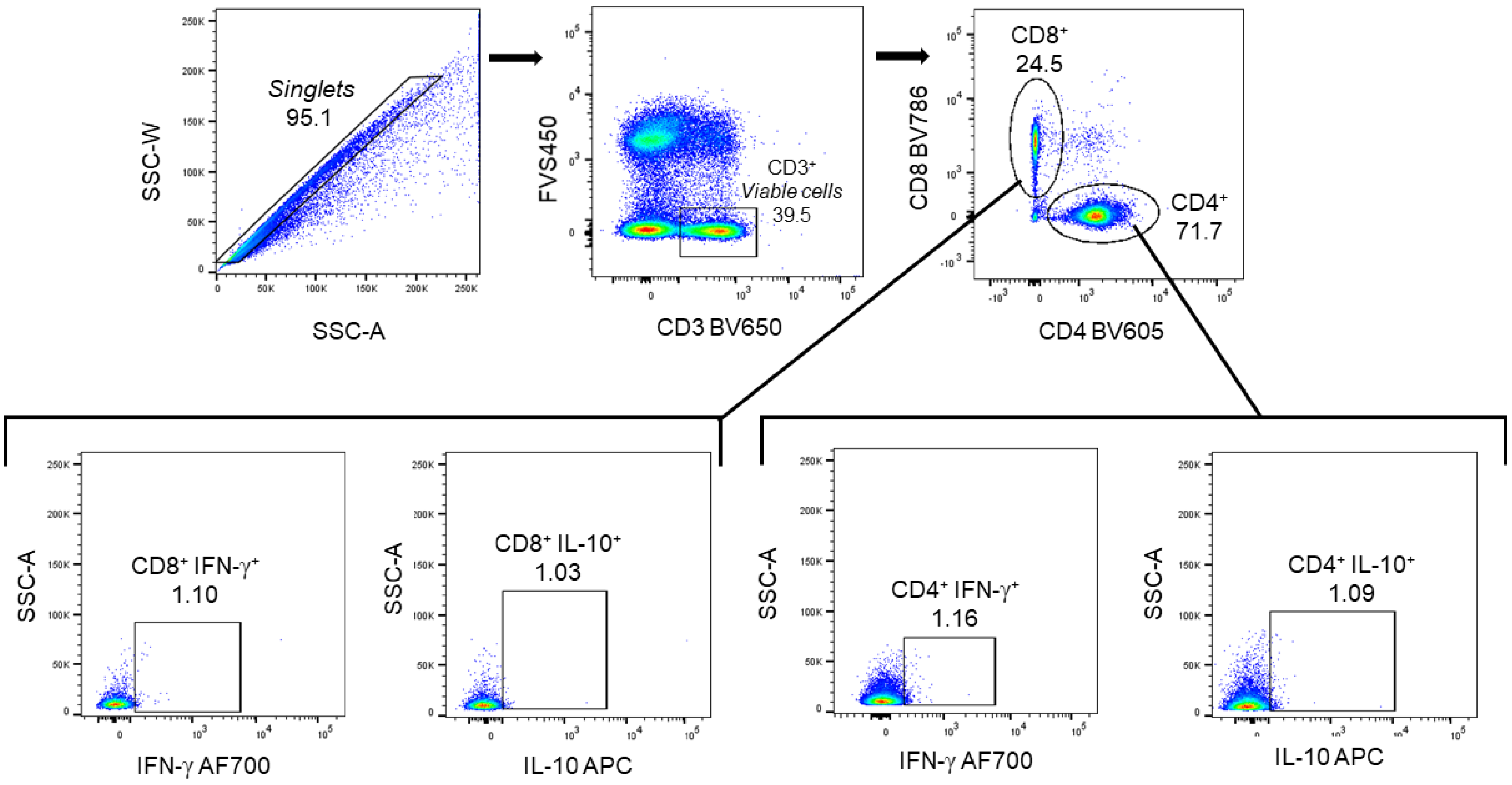

Supplement: Supplementary file 1 [file biology-12-00851-s001.zip › biology-2357597-supplementary.pdf]
